# Lessons Learned from Surveillance of Antimicrobial Susceptibilities of *Pseudomonas aeruginosa* at a Large Academic Medical Center ^†^

**DOI:** 10.3390/ph3041070

**Published:** 2010-04-01

**Authors:** Brett H. Heintz, Jenana Halilovic

**Affiliations:** 1 Clinical Pharmacy, San Francisco School of Pharmacy, University of California, 521 Parnassus Avenue UCSF Box 0622, Room C-152 San Francisco, CA 94143, USA; 2 Department of Pharmaceutical Services, University of California Davis Health System, Sacramento, CA 95817, USA; 3 Thomas J. Long School of Pharmacy and Health Sciences, University of the Pacific, 3601 Pacific Avenue, Stockton, CA 95211, USA; E-Mails: JHalilovic@Pacific.edu (J.H.)

**Keywords:** *Pseudomonas aeruginosa*, antimicrobials, antibiotics, bacterial resistance, cefepime, antimicrobial stewardship, antibiogram

## Abstract

This research report assessed the differences in resistance rates and antimicrobial usage-versus-susceptibility relationships of *Pseudomonas aeruginosa* found in various hospital patient care areas. A simplified case control study was also performed to identify patient-specific risk factors associated with cefepime-resistant *P. aeruginosa* isolates. Last, we determined the consequence of combining mucoid and non-mucoid derived antimicrobial susceptibilities of *P. aeruginosa* into hospital antibiograms. Overall, susceptibility rates remained lower in the intensive care units (ICUs) compared to the non-ICU patient care areas, except for cefepime over the last time period. Cefepime utilization and antimicrobial-resistance rates among *P. aeruginosa* isolates had a significant relationship. Decreased meropenem exposure was associated with lower resistance rates relative to cefepime. Risk factors independently associated with cefepime-resistant *P. aeruginosa* were structural lung disease, ICU admission, recent third generation cephalosporin use, frequent hospital admission and non-urine isolates. Large and statistically significant differences were observed between non-mucoid and combined percent susceptibility data for aminoglycosides. To control antimicrobial resistance and optimize initial empiric antimicrobial therapy, antimicrobial susceptibility and utilization patterns in specific patient care areas should be monitored and risk factors for antimicrobial resistance should be assessed. Mucoid strains of *P. aeruginosa* should not be included into antimicrobial susceptibility data as this may underestimate activity of most antipseudomonal agents.

## 1. Introduction

*Pseudomonas aeruginosa*, an emerging nosocomial pathogen, is characterized as an aerobic, lactose negative, oxidase positive, and slightly curved gram-negative rod with varied morphology (e.g., non-mucoid variants and less commonly mucoid variants associated with cystic fibrosis) [1]. The high mortality associated with *P. aeruginosa* infections, particularly with ineffective initial empiric therapy, emphasizes the need for reliable data on which to base the choice of empiric therapy [2]. Significant declines in the susceptibility of *P. aeruginosa* to many antimicrobials were noted at our institution, primarily for cefepime, ciprofloxacin and tobramycin. Most alarming was the rapidly increasing resistance rates of *P. aeruginosa* to cefepime, which is considered to be the first-line antimicrobial agent for empiric nosocomial gram-negative-rod coverage at our institution. Optimal control and treatment of *P. aeruginosa* infections traditionally have been a focus of antimicrobial stewardship programs. Cefepime is currently approved for intensive care unit (ICU) empiric therapy when *P. aeruginosa* is suspected, while carbapenems require approval by the antimicrobial stewardship team. Although multiple factors play a role in the increased resistance rates, the selective pressure of inappropriate and increased antimicrobial utilization are considered major contributors [3]. Current evidence suggests that controlling antimicrobial resistance requires monitoring susceptibility trends and monitoring and modifying antimicrobial usage within specific patient care areas of the hospital [4]. 

The primary objective of this retrospective study was to assess the differences in antipseudomonal resistance rates and antimicrobial usage-versus-susceptibility relationships of *P. aeruginosa* found in various patient care areas of the hospital. Secondary objectives were to determine the consequence of combining mucoid-cystic fibrosis and non-mucoid derived antimicrobial susceptibilities of *P. aeruginosa* into hospital antibiograms and to identify patient-specific risk factors associated with cefepime-resistant *P. aeruginosa* isolates for non-ICU patients. 

## 2. Methods

The University of California, Davis Medical Center (UCDMC) is a 613 bed tertiary care teaching hospital located in Sacramento, California. Census, antimicrobial usage, and susceptibility data were collected semiannually and retrospectively from July 2000 through June 2006 to assess the differences between antimicrobial use-versus *P. aeruginosa* susceptibility relationships found in the various patient care areas of the hospital. Antimicrobial susceptibility data for the following patient care areas within the University of California, Davis Health System (UCDHS) were included in the study: adult non-ICU inpatient care areas collectively, adult ICUs [medical ICU (MICU), surgical/trauma ICUs (SICUs), medical-surgical ICU (MSICU), neurosurgical ICU (NICU), coronary care unit (CCU) and the burn unit], adult specialty patient care areas (hematology/oncology wards and kidney transplant unit), and outpatient care areas collectively. The total patient-days of hospitalization for a given time period for the individual patient care areas were obtained from the hospital admissions department. This study was approved by our institutional review board.

### 2.1. Antimicrobial Usage

Total grams of inpatient antimicrobials purchased were electronically transferred from the hospital pharmacy computer system to a Microsoft excel spreadsheet. Utilization data for specific patient care areas was available for cefepime, meropenem, piperacillin, ciprofloxacin and tobramycin (grams dispensed) from July 2005 through December 2005. These data were used to express normalized antimicrobial drug use in defined daily doses (DDD) per 1,000 patient days (DDD/1,000 PD) as recommended by the World Health Organization (WHO) Collaborating Centre for Drug Statistics Methodology [5]. A DDD is the assumed average maintenance dose per day for a drug used for its primary indication in adults [5]. A DDD of three grams was used for cefepime, two grams for meropenem, 14 grams for piperacillin (piperacillin-tazobactam included), 800 mg for ciprofloxacin and 300 mg for tobramycin. Cefepime and tobramycin DDD differed from those recommended by WHO (three grams *vs.* two grams and 300 mg *vs.* 240 mg, respectively) to reflect normalized dosing recommendations within our institution. At least 20 isolates per patient care area were required for inclusion in the unit-specific statistical analysis. 

### 2.2. Susceptibility Data

The surveillance network (TSN) database was used as the source of antimicrobial susceptibility testing for this study. The surveillance network electronically assimilates antimicrobial susceptibility testing results and patient demographic data for our institution, among other network hospitals. Only non-urine and non-repeat isolates of *P. aeruginosa* were included for review. Semiannual UCDMC susceptibilities were determined from July 2000 to June 2006 for cefepime, ceftazidime, ciprofloxacin, gentamicin, meropenem, piperacillin and tobramycin. Distributions of cefepime minimum inhibitory concentrations (MICs) for *P. aeruginosa* were determined using the TSN database from January 2005 to June 2006. The susceptibility breakpoints used by the system were in accordance with guidelines of the Clinical and Laboratory Standards Institute (CLSI) during the study period. Data was collected retrospectively and semiannually. Neither the susceptibility testing methods nor the susceptibility breakpoints changed during the study period. All percentages are expressed as absolute percentages. Time series analysis was utilized to express the susceptibility data. 

During the exploratory phase of the study it was discovered that mucoid *P. aeruginosa* strains were included into susceptibility reporting between July 2005 and June 2006. This was a major finding of this study as it coincided with implementation of the electronic medical record (EMR) system at UCDMC. Antimicrobial susceptibilities of mucoid and combined mucoid and non-mucoid *P. aeruginosa* isolates were available from the TSN database. Cefepime, ceftazidime, ciprofloxacin, meropenem, piperacillin, amikacin, gentamicin and tobramycin susceptibilities of non-mucoid *P. aeruginosa* were determined by calculation and were compared to combined susceptibilities over the last study period (January–June 2006).

### 2.3. Simplified Case Control Study

We observed a visual trend of higher *P. aeruginosa* resistance rates to cefepime for non-ICU patients compared to ICU patients, collectively, for the last time-period (January 2006–June 2006). As a result, a simplified (retrospective, non-matched) case-control study was performed to define various patient-specific risk factors for cefepime-resistant (intermediate or fully resistant, MIC > 8 mg/L) *P. aeruginosa* among non-ICU patients. During this time period, patients in non-ICU patient care areas with cefepime-resistant *P. aeruginosa* isolates were compared to those with susceptible strains. The following parameters were analyzed: patient demographic data, patient care service at the time isolate was recorded, initial ICU admission, past medical history per initial inpatient admission summary, history of present illness, source of *P. aeruginosa* isolate, *P. aeruginosa* strain (mucoid *vs.* non-mucoid), third generation cephalosporin use within the last 90 days, assessment of multidrug-resistance (resistance to ≥ three antipseudomonal agents), length of stay, number of admissions in the last six months, and selected chronic diseases. Chronic diseases of interest were uncontrolled-symptomatic cardiovascular disease (heart failure or coronary heart disease), obstructive lung disease (chronic obstructive pulmonary disease and asthma), absolute or functional neutropenia (absolute neutrophil count < 1,000 cells/mm^3^), prednisone equivalent > 10 mg daily for at least the last seven days, solid organ or hematologic transplantation, solid organ malignancy, HIV/AIDS, diabetes mellitus, hemodialysis use in the last 30 days, end stage liver disease, and structural lung disease (e.g. cystic fibrosis or bronchiectasis). The same patient was only included if *P. aeruginosa* isolates were separated by at least seven days. This follow-up observational study was approved by our institutional review board.

### 2.4. Statistical Analysis

Continuous variables were compared using the Student’s t-test for normally distributed variables and the Mann-Whitney test for non-normally distributed variables. Chi-square or Fishers exact tests, as appropriate, were used to compare categorical variables. All comparisons were unpaired, all tests were two-tailed unless otherwise specified, and p-values (p) of < 0.05 were considered statistically significant. All percentages are expressed as absolute percentages unless otherwise specified. Analyses were completed using Minitab® statistical software (version 13, State College, PA).

To find the relationship between cefepime utilization in specific patient care areas and cefepime-resistant *P. aeruginosa* rates from July 2000 to June 2006, a linear regression analysis was performed and a Pearson’s correlation coefficient (R) and corresponding p-values (one-tailed) were determined using the Student’s t-test. The same methodology was utilized to find the relationship of meropenem, piperacillin, ciprofloxacin, and tobramycin utilization and corresponding resistance rates among *P. aeruginosa* isolates. A chi-square analysis or Fisher’s exact test with Bonferroni correction, as appropriate, were utilized to compare antimicrobial susceptibilities of combined mucoid and non-mucoid *P. aeruginosa* to calculated non-mucoid *P. aeruginosa* isolates. Corresponding p-values were determined. To determine independent predictors for cefepime-resistant *P. aeruginosa* isolates, univariate logistic and multivariate stepwise (unconditional forward and backward) regression analyses were performed. Variables significant at p < 0.20 in the univariate analysis were entered into the model in a stepwise fashion with a p-value threshold of 0.10 for acceptance or removal of variables. Odds ratios (OR) with 95% confidence intervals (CI) and corresponding p-values were subsequently determined. 

## 3. Results

**Figure 1:**
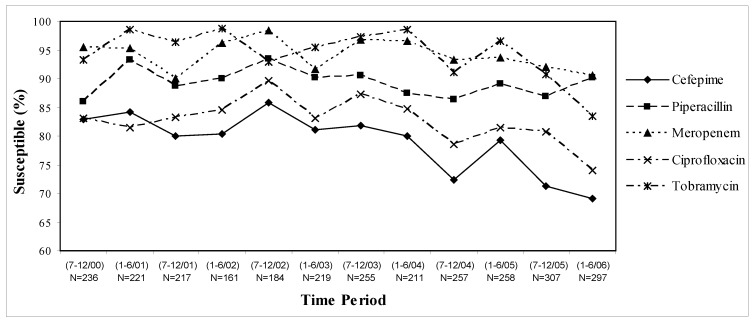
UCDMC antimicrobial susceptibilities of *P. aeruginosa*: July 2000–June 2006.

Overall, antimicrobial susceptibility rates among *P. aeruginosa* isolates decreased by 10–15% over the six year study period (Figure 1), however susceptibilities varied considerably by patient-care areas (data not shown). Pooled inpatient and outpatient data indicates that piperacillin and meropenem were the most active and stable against *P. aeruginosa* with approximately 90% susceptibility rates. In fact, rates of susceptibilities to meropenem decreased by only 5%, while piperacillin susceptibility rates increased by 5% over the whole study period. In comparison, susceptibility rates to cefepime have decreased by 15% over the six year study period with a 10% decrease over the last two years alone. While ciprofloxacin susceptibility rates decreased by 10% over the study period, tobramycin susceptibilities remained stable from 2000 through early 2005, but then decreased by 15% over the last year alone (Figure 1). 

**Figure 2:**
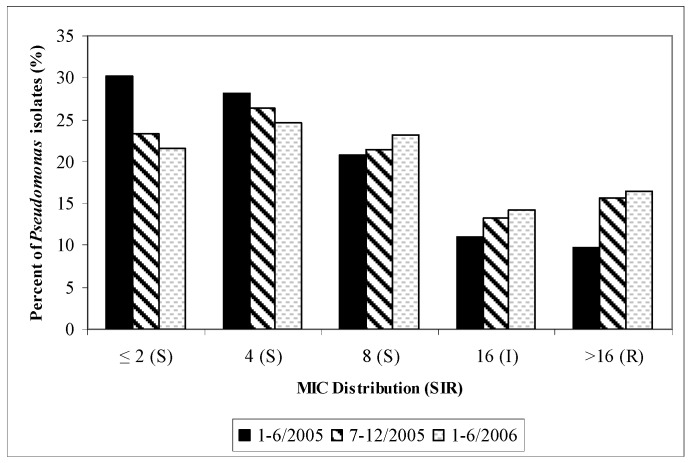
Cefepime susceptibilities of *P. aeruginosa* (MIC Distribution): UCDMC (semiannually, January 2001–June 2006).

Antimicrobial susceptibility rates remained lower in the ICUs compared to the non-ICU patient care areas, except cefepime over the last time period (January–June 2006, data not shown). In the outpatient analysis, it was found that tobramycin-susceptibilities of *P. aeruginosa* decreased by 32% (100% to 68%) from January 2004–June 2006 and the number of isolates have nearly quadrupled (n = 40 to n = 148) over the last study year. Further, it was determined that gentamicin and amikacin susceptibilities of *P. aeruginosa* decreased by 47% (95% to 48%) and 43% (100% to 57%), respectively, during the same time period. After consulting with the microbiology department and review of specific patient cases it was determined that mucoid strains of *P. aeruginosa* were included in the TSN database since mid 2005. A visual trend in cefepime MIC shift (“MIC creep”) and cefepime non-susceptibility (MIC > 8mg/L) to *P. aeruginosa* were found with time (Figure 2). 

**Figure 3:**
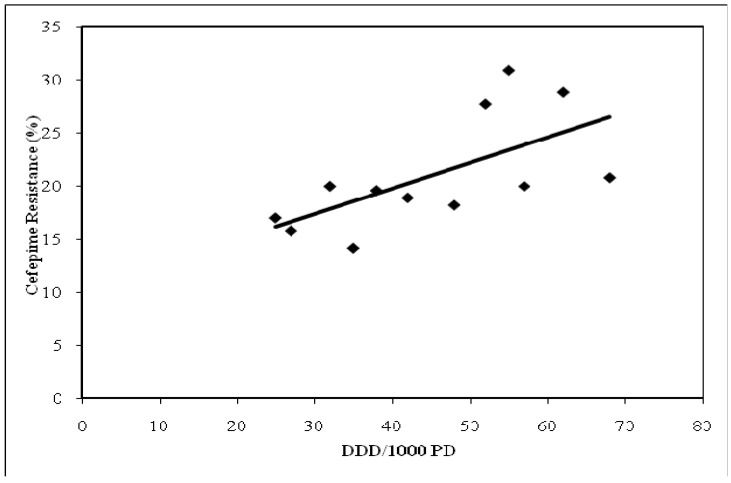
Relationship between cefepime utilization and resistance among P. aeruginosa isolates: July 2000–June 2006, UCDMC (R = 0.64), P = 0.013.

**Figure 4:**
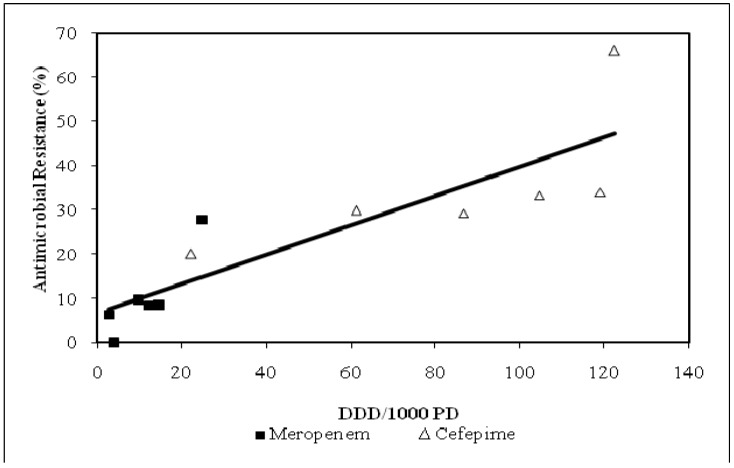
Relationship between cefepime *vs.* meropenem utilization and resistance among *P. aeruginosa* isolates: July-December 2005, Unit-specific (R = 0.87, p < 0.001).

**Figure 5:**
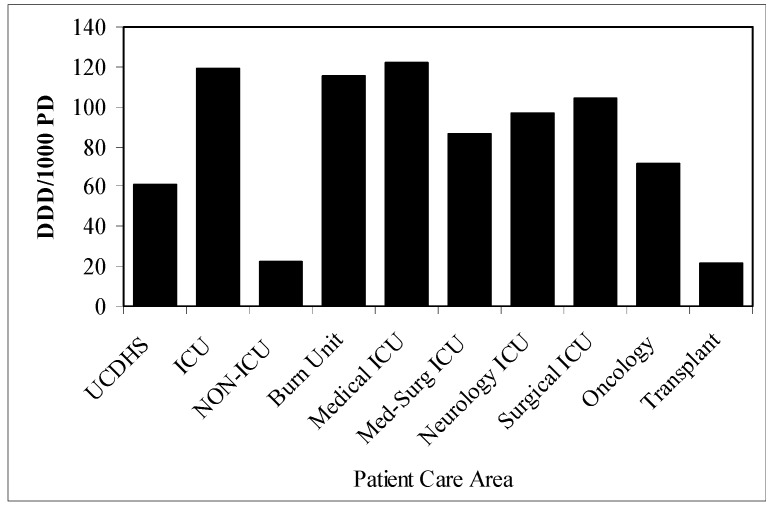
Cefepime utilization (defined daily dose per 1000 patient days = DDD/1000 PD) by patient care area.^ a^

^a^ Non-ICU excludes oncology and transplant patient-care areas.

Cefepime semiannual UCDMC usage over the six year study period and corresponding *P. aeruginosa* susceptibilities had a significant relationship (R = 0.64, p = 0.013, Figure 3). Cefepime usage and *P. aeruginosa* susceptibility trended towards significance defined by collective inpatient care areas and when individual ICUs were compared (R = 0.71, p = 0.054 and R = 0.91, p = 0.13, respectively; data not shown). Meropenem usage and *P. aeruginosa* susceptibility had a significant relationship defined by collective inpatient care areas and when individual ICUs were compared (R = 0.90, p = 0.012 and R = 0.99, p = 0.036, respectively; data not shown). Decreased meropenem exposure was associated with lower resistance rates relative to cefepime (R = 0.87, p < 0.001; Figure 4). The mean use of cefepime (DDD/1000 PD) increased from 25.1 to 53.3 (112.4%) from 2000 to 2006, respectively. Utilization of cefepime in adult ICUs was approximately five times that of adult non-ICUs and varied considerably by patient care area (Figure 5). 

Antimicrobial susceptibilities varied significantly when non-mucoid and combined isolates of *P. aeruginosa* were compared. For example, large and statistically significant differences were observed for gentamicin and amikacin susceptibilities (82.5% *vs*. 65.3%, p < 0.001; 95.8% *vs.* 76.4%, p < 0.001, respectively), but not for β-lactams, tobramycin or ciprofloxacin (Table 1). In addition, statistically significant differences were noted comparing antipseudomonal susceptibilities of non-mucoid and mucoid *P. aeruginosa* isolates for most agents (data not shown), except for meropenem and tobramycin which maintained excellent and stable activity against both non-mucoid and mucoid strains of *P. aeruginosa* (90.5% *vs.* 90.7%, NS and 85.2% *vs.* 80.6%, NS, respectively). Of interest, piperacillin and gentamicin had similar activity relative to meropenem and tobramycin, respectively, for non-mucoid strains of *P. aeruginosa* (Table 1).

Among the analysis of non-ICU patients with positive *P. aeruginosa* isolates, 20 were non-susceptible to cefepime (18 patients) and 57 were susceptible (50 patients). Multi-drug resistant *P. aeruginosa* was found in sixteen of the cefepime-resistant isolates (80%). Risk factors for non-susceptible *P. aeruginosa* isolates to cefepime included: structural lung disease (OR = 5.6, p = 0.009), mucoid strains of *P. aeruginosa* (OR = 7.13, p = 0.005), diabetes (OR = 3.07, p = 0.044), initial ICU admission (OR = 5.7, p = 0.015), third generation cephalosporin use within six months (OR = 5.5, P = 0.006), and ≥ two admissions in the last six months (OR = 9.90, p < 0.001) upon univariate logistic regression (Table 2). However, diabetes and mucoid strains were not found to be independent predictors of cefepime-resistance upon multivariate stepwise regression. Comparing patients with fully-resistant isolates to patients with susceptible isolates provided similar results (data not shown). End stage liver disease appeared to increase the risk for cefepime-resistant *P. aeruginosa* isolates, although this finding was not statistically significant. Immunocompromised state, hemodialysis in the last 30 days, obstructive lung disease, and cardiac disease did not affect *P. aeruginosa* susceptibilities to cefepime (Table 1). Unexpectedly, patients 60 years or older appeared to be at decreased risk for cefepime-resistant *P. aeruginosa* isolates, although not statistically significant upon multivariate regression analysis.

MIC = minimum inhibitory concentration: S = susceptible; I = Intermediate; R = resistant

DDD/1000 PD = defined daily dose per 1000 patient days

DDD/1000 PD = defined daily dose per 1000 patient days.

DDD/1000 PD = defined daily dose per 1000 patient days, UCDHS = UC Davis Health System, ICU = intensive care unit.

**Table 1:** Differences between non-mucoid and combined percent susceptible data.

Antimicrobial Agent	All *PA* isolates (n = 297) % Susceptible	Mucoid *PA* (n = 108) % Susceptible	Non-mucoid (n = 189) % Susceptible	Chi-Square Analysis p-value^a^
Cefepime	69.1	61.1	73.5	0.271
Ceftazidime	84.8	78.7	87.9	0.307
Piperacillin	90.2	82.4	94.7	0.077
Meropenem	90.6	90.7	90.5	1.00
Ciprofloxacin	74	64.8	79.4	0.181
Gentamicin	65.3	34.3	82.5	<0.001
Amikacin	76.4	42.6	95.8	<0.001
Tobramycin	83.4	80.6	85.2	0.617

^a^ Comparing combined *P. aeruginosa* susceptibilities to non-mucoid *P. aeruginosa.*

**Table 2:** Risk Factors for cefepime-resistant (intermediate or fully-resistant) *Pseudomonas aeruginosa* isolates (non-ICU adult patients, January-June 2006).

Condition	Cefepime Resistant n = 20, n (%)	Cefepime Susceptible n = 57, n (%)	OR (95% CI) Univariate Analysis	P-value (2-tailed)
Mucoid strain	7 (35)	4 (7)	7.13 (1.81-28.08)	p = 0.005
Structural lung disease	7 (35)	5 (9)	5.60 (1.53-20.52)	p = 0.009^a^
Diabetes mellitus	9 (45)	12 (21)	3.07 (1.03-9.10)	p = 0.044
Immunosuppression	9 (45)	17 (30)	1.93 (0.68-5.49)	p = 0.221
Hemodialysis in last 30 days	1 (5)	4 (7)	0.69 (0.07-6.64)	p = 0.627
Obstructive lung disease	4 (20)	14 (25)	0.70 (0.20-2.43)	p = 0.574
End stage liver disease	2 (10)	3 (5)	4.85 (0.75-31.49)	p = 0.098
Cardiac disease	2 (10)	14 (25)	0.16 (0.02-1.32)	p = 0.089
No risk factors identified	1 (5)	9 (16)	0.28 (0.03-2.37)	p = 0.243
Non-urine isolates	19 (95)	23 (40)	13.30 (2.81-62.92)	p = 0.001^a^
Initial ICU admission	6 (30)	4 (7)	5.68 (1.41-22.93)	p = 0.015^a^
TGC use within 90 days	14 (70)	22 (38)	4.00 (1.33-11.99)	p = 0.013^a^
Length of admission ≥ 5 days	17 (85)	40 (70)	2.21 (0.57-8.59)	p = 0.252
≥ 2 admissions over last 6 months	13 (65)	10 (18)	9.90 (3.10-31.67)	p < 0.001^a^
Age ≥ 60 years old	2 (10)	24 (42)	0.15 (0.03-0.72)	p=0.018

OR = Odds ratio, CI = Confidence interval, ICU = intensive-care unit, TGC = Third generation cephalosporin (e.g. ceftriaxone, cefotaxime and ceftazidime). ^a^ Statistically significant (p < 0.05) upon stepwise multivariate regression; data not shown.

## 4. Discussion

Resistance to antimicrobial agents is an increasing public health threat. It limits therapeutic options and leads to increased morbidity and mortality [6]. Hospital wide surveillance has traditionally been used for the detection of resistance problems within an institution, such as our own. However, such reliance on hospital-wide surveillance data may be misleading since these data can mask important antimicrobial resistance trends within individual patient care areas of the hospital due to variability in patient populations, antimicrobial use, and infection-control practices within a given institution [3,7,8,9,10]. For example, data from our institution indicates that adult ICUs have greater antimicrobial resistance rates of *P. aeruginosa* for most agents compared to non-ICUs, likely due to a higher risk patient population and increased antimicrobial utilization. Among the individual ICUs, the SICU had significantly lower rates of *P. aeruginosa* resistance compared to other adult ICUs, likely due to a younger and lower risk patient population as well as more appropriate antimicrobial utilization. For instance, the SICU team routinely utilizes clinical pulmonary infection scores (CPIS) to guide appropriate antimicrobial utilization and were initiated as standard practice in July 2004 (personal communication). Review of SICU antimicrobial susceptibilities of *P. aeruginosa* comparing the first half of 2004 to the first half of 2006 resulted in clinically and statistically significant differences for cefepime (60.0% to 86.7%, p = 0.045) and ciprofloxacin (65.1% to 93.3%, p = 0.032) and clinically, but non-statistically significant differences for meropenem (81.8% to 100%, p = 0.082) and tobramycin (83.7% to 100%, p = 0.10) using a one-tailed chi-square analysis. Thus, utilization of infection scoring systems to guide antimicrobial therapy may help control the spread of antimicrobial resistance.

The burn-unit represents another example where utilization of unit-specific antibiograms reveals important antimicrobial susceptibility patterns. Meropenem susceptibilities dropped nearly 40% in the burn unit during the last time period (January–June 2006). This was likely due to an *Acinetobacter* outbreak during this time period resulting in increased meropenem utilization, the drug of choice and most active agent against *Acinetobacter* species at our institution. Interestingly, piperacillin susceptibilities in the burn unit steadily improved and increased 36% over the 2.5 year study period. These important susceptibility findings are limited by the small number of isolates. Nonetheless, development of unit-specific antibiograms may play an important role in guiding empiric selection of antimicrobial therapy.

As a result of this study, unit-specific antibiograms are being developed at our institution. Utilizing unit-specific antibiograms will increase the likelihood of initial adequate empiric therapy and lead to more appropriate antimicrobial utilization and possibly improved patient outcomes [9]. When deciding on an antimicrobial regimen, including the need to empirically double cover *Pseudomonas*, if suspected, clinicians must consider individual patient care area susceptibility patterns (e.g., ICU *vs.* non-ICU), the patients’ clinical status (e.g., critically ill *vs.* clinically stable) and the patients’ inherent risk factors (e.g., structural lung disease and/or risk for mucoid strains of *P. aeruginosa vs.* non-mucoid *P. aeruginosa*).

Cefepime and meropenem usage and susceptibility to *P. aeruginosa* had a significant relationship, especially when individual ICUs were compared. Decreased meropenem exposure was associated with lower resistance rates relative to cefepime. Cefepime use more than doubled from 2000 to 2006, whereas resistance rates of *Pseudomonas* increased to a lesser degree (15%). However, studies indicate that individual patient exposures to specific antimicrobials may drive resistance [11]. For instance, it was shown in our study that third-generation cephalosporin exposure was a strong predictor of cefepime-resistant *P. aeruginosa*. Group level and individual patient level analysis of antimicrobial use *vs.* susceptibility relationships would likely yield divergent results. The epidemiologic relationship between antimicrobial use and resistance is complex and requires further study. 

UCDHS cefepime utilization is approximately 2.5 times the national average (DDD/1000PD = 53.3 *vs.* 21.9); however piperacillin/tazobactam and ceftazidime utilization are about one-third and one-half of the national average, respectively (DDD/1,000 PD = 10.25 *vs.* 30.3; 5.1 *vs.* 9.9, respectively) [4,12]. Given the high cefepime utilization, reduced cefepime susceptibilities of *Pseudomonas* and cefepime MIC distribution confirming MIC “creep” with time, we considered changing our recommended empiric antipseudomonal β-lactam for high risk ICU patients (and selected non-ICU patients) from cefepime to piperacillin/tazobactam. However, it has recently been shown that increased utilization of piperacillin/tazobactam increases cefepime resistance to *Enterobacter cloacae* and *E. aerogenes*, *Klebsiella pneumoniae, Eschericia coli* and *Pseudomonas aeruginosa* [12]. Thus, antimicrobial cycling at our institution (e.g. changing cefepime to piperacillin-tazobactam as the recommended empiric antipseudomonal β-lactam) may not be an effective approach to recover cefepime susceptibility. Further, pooled national susceptibility data indicates that cefepime is collectively more active than piperacillin/tazobactam and ceftazidime against clinically important Enterobactericiae and *P. aeruginosa* isolates [12]. At our institution cefepime has excellent activity against most clinically important aerobic Gram-negative rod nosocomial pathogens (e.g., >95% susceptibility, data not shown) except for *Acinetobacter*, *Stenotrophomonas* and *Pseudomonas* species. Thus, it was decided that a better method of controlling *P. aeruginosa* resistance to cefepime is through improved cefepime utilization. UCDHS inpatient cefepime guidelines are being revised as a result of this study.

It is unclear why non-ICUs had higher rates of cefepime-resistant *P. aeruginosa* isolates compared to ICUs over the last time period. All inpatient mucoid *P. aeruginosa* isolates (n = 11, eight patients) were isolated from non-ICU patients with cefepime susceptibility rates of 27.2%. However, given the small number of mucoid isolates it did not statistically alter combined *vs.* non-mucoid susceptibilities to cefepime for non-ICU patient care areas (55.6% *vs.* 68.4%, respectively, p = 0.28) and was still lower than collective ICU *P. aeruginosa* susceptibilities to cefepime (75.6%). After analysis of non-ICU patients during the last study period it was found that non-urine isolates, structural lung disease, third generation cephalosporin exposure, initial ICU admission and recent and frequent hospital admissions were independent predictors of resistant *P. aeruginosa* isolates to cefepime. Some unexpected findings were noted as well. For example, length of admission was not found to be an independent predictor of *P. aeruginosa* resistance to cefepime. Also, patients 60 years or older was associated with protection against cefepime resistance upon univariate analysis, however this finding did not hold true upon multivariate analysis. Among patients 60 years or older, 19 of 24 patients (79.2%) in the susceptible group had *P. aeruginosa* isolated from urine and 23 of 24 patients (95.8%) were non-mucoid strains. This may possibly explain the appeared “protection” against resistance upon univariate analysis as our study found non-urine sources and mucoid isolates to be predictive of cefepime resistance (Table 2). 

Recent advances in hospital informatics systems and surveillance software have increased the ability of hospital computer systems to gather hospital data. These systems have the capability of providing pharmacy and susceptibility data which may provide a more efficient antimicrobial surveillance program. However, new technology brings challenges that must be resolved. At our institution, after implementation of EMR, it was determined from the exploratory phase of this study that mucoid strains of *P. aeruginosa* were included into the TSN susceptibility database and were reflected into current hospital-wide antibiogram data, likely contributing to rapidly reduced *P. aeruginosa* susceptibilities to various antipseudomonal agents in the last year of the study (e.g., 30%–45% reduction in susceptibilities to aminoglycosides and four-fold increase in the number of *P. aeruginosa* isolates were noted for outpatient care areas). Combining mucoid and non-mucoid *P. aeruginosa* isolates into hospital antibiograms produce figures that underestimate the activity of some antimicrobial classes, primarily aminoglycosides and fluoroquinolones, and thus may alter initial empiric antimicrobial selection [13]. 

Meropenem and tobramycin were the most stable and active antimicrobials against mucoid strains of *P. aeruginosa*. Amikacin was the most active aminoglycoside for non-mucoid strains of *P. aeruginosa*, while gentamicin and tobramycin had similar susceptibilities for non-mucoid strains (Table 1). Thus, combination of meropenem and tobramycin would be the most active initial empiric regimen for mucoid strains of *P. aeruginosa* at our institution. An antipseudomonal β-lactam in combination with gentamicin would be appropriate initial empiric therapy for suspected non-mucoid strains. Upon availability of susceptibility results, streamlined β-lactam monotherapy can be utilized for most patients, as recent data has showed no benefit to combination therapy once susceptibilities were known (except for patients with *P. aeruginosa* bloodstream infections and/or neutropenia), however further discussion is beyond the scope of this review [14,15,16]. Piperacillin should be utilized for definitive therapy of *P. aeruginosa* strains highly susceptible to piperacillin, if possible, as this may prove to be more cost-effective through lower utilization costs, equal efficacy when dosed appropriately and the ability for continuous infusion dosing for severe infections and/or outpatient therapy, while possibly recovering meropenem susceptibility to *Pseudomonas* by taking the pressure off meropenem.

This study was not without limitations. Our institution has utilized the Phoenix automated susceptibility system for antimicrobial susceptibility reporting since 2005. Recently, it has been shown that the Phoenix system may under report *P. aeruginosa* susceptibilities to cefepime and may partially explain reduced *P. aeruginosa* susceptibilities over the last two years [17]. Time series statistical analysis to test the significance of susceptibility trends was not performed, except for the SICU. To test for a possible trend, a chi-square or Mantel Henzel test, as appropriate, could have been performed. While hospital-wide cefepime utilization was available from 2000–2006, only six months of data were available for the unit-specific assessment of cefepime and meropenem use. Perhaps, unit-specific antimicrobial utilization available for all time periods would have provided divergent results. Defined daily doses for other antimicrobials were determined; however, data was incomplete and unreliable and were not included in this review. UCDMC cefepime utilization was extracted from pharmacy purchasing data for the six year utilization review. Ideally, grams of antimicrobial doses received or dispensed would have been more accurate methodologies to calculate DDD/1000 PD, although this data was not available, except for the unit-specific analysis performed from July-December 2005. The combined *vs.* non-mucoid susceptibilities were only assessed for the most recent complete six months of data (January–June 2006) and it is possible that results would have differed if other time periods were analyzed. 

The simplified case-control study identified predictors for *P. aeruginosa* resistance to cefepime among non-ICU patients, however was limited by many factors, including a small sample size which may limit reliability. It is possible that other variables neither accounted for nor included into the model may have altered the results upon multivariate regression analysis. For example, severity of illness, indwelling catheters, invasive procedures and devices, skilled nursing facility residence and home infusion therapy have also been associated with *P. aeruginosa* resistance in the literature [18]. Conversely, it is possible that too many variables were included in the multivariate regression analysis which may limit reliability. Further, an assessment of predictors of resistance to other antimicrobials and for multi-drug resistance was not determined. Also of interest would have been a case control study comparing the group of patients with *P. aeruginosa* infections (cases) to a randomized matched control group to assess predictors for *P. aeruginosa* infections. This would allow for better optimization of initial empiric therapy by identifying patients at risk for *Pseudomonas* infections and possibly drug-resistant *Pseudomonas* infections based on defined risk factors. Perhaps variables that did not predict resistance may have been linked to *P. aeruginosa* infections in general, such as advanced age. 

## 5. Conclusions

In order to control the spread of antimicrobial resistance, the pattern of antimicrobial susceptibilities and utilization in specific patient care areas should be monitored and may lead to improved initial empiric therapy. In our non-ICU patient population, cefepime resistance was associated with patient-specific risk factors. Effective initial empiric antimicrobial therapy requires consideration of identified risk factors for *P. aeruginosa* resistance. In our adult ICU patient population, increased cefepime exposure was associated with decreased activity against *P. aeruginosa*. Carbapenem resistance has remained low primarily due to decreased utilization through antimicrobial approval policies and stewardship. A multidisciplinary approach is necessary to succeed in curbing resistance. Lastly, mucoid strains should not be included into antimicrobial susceptibility data as this may underestimate activity of most antipseudomonal agents and likely alter prescribing patterns.
